# Effects of acclimation temperature and feed restriction on the metabolic performance of green sturgeon

**DOI:** 10.1093/conphys/coae021

**Published:** 2024-05-06

**Authors:** Kenneth W Zillig, Kelly D Hannan, Sarah E Baird, Dennis E Cocherell, Jamilynn B Poletto, Nann A Fangue

**Affiliations:** Department of Wildlife, Fish, and Conservation Biology, University of California Davis, One Shields Avenue, Davis, CA 95616-5270, USA; Department of Wildlife, Fish, and Conservation Biology, University of California Davis, One Shields Avenue, Davis, CA 95616-5270, USA; Department of Wildlife, Fish, and Conservation Biology, University of California Davis, One Shields Avenue, Davis, CA 95616-5270, USA; Department of Wildlife, Fish, and Conservation Biology, University of California Davis, One Shields Avenue, Davis, CA 95616-5270, USA; Department of Wildlife, Fish, and Conservation Biology, University of California Davis, One Shields Avenue, Davis, CA 95616-5270, USA; Department of Wildlife, Fish, and Conservation Biology, University of California Davis, One Shields Avenue, Davis, CA 95616-5270, USA

**Keywords:** Aerobic scope, Chinook salmon, conservation conflict

## Abstract

Green sturgeon (*Acipenser medirostris*) are an anadromous threatened species of sturgeon found along the Pacific coast of North America. The southern distinct population segment only spawns in the Sacramento River and is exposed to water temperatures kept artificially cold for the conservation and management of winter-run Chinook salmon (*Oncorhynchus tshawytscha*). Past research has demonstrated costs of cold-water rearing including reduced growth rates, condition and survivorship of juvenile green sturgeon. Our research investigates how the stressors of water temperature and food limitation influence the metabolic performance of green sturgeon. We reared green sturgeon at two acclimation temperatures (13 and 19°C) and two ration amounts (100% and 40% of optimal feed). We then measured the routine and maximum metabolic rates (RMR and MMR, respectively) of sturgeon acclimated to these rearing conditions across a range of acute temperature exposures (11 to 31°C). Among both temperature acclimation treatments (13 or 19°C), we found that feed restriction reduced RMR across a range of acute temperatures. The influence of feed restriction on RMR and MMR interacted with acclimation temperature. Fish reared at 13°C preserved their MMR and aerobic scope (AS) despite feed restriction, while fish fed reduced rations and acclimated to 19°C showed reduced MMR and AS capacity primarily at temperatures below 16°C. The sympatry of threatened green sturgeon with endangered salmonids produces a conservation conflict, such that cold-water releases for the conservation of at-risk salmonids may constrain the metabolic performance of juvenile green sturgeon. Understanding the impacts of environmental conditions (e.g. temperature, dissolved oxygen) on ecological interactions of green sturgeon will be necessary to determine the influence of salmonid-focused management.

## Teaser

We reared green sturgeon at two temperatures and provided two rations. We then assessed the metabolic performance of fish from these four treatments across a range of temperatures (11 to 31#x00B0;C). The impacts of temperature and ration create a conservation conflict between green sturgeon and the endangered winter-run Chinook salmon.

## Introduction

Temperature plays a fundamental role in the biology of organisms across biological scales (Brett, 1952; Hochachka and Somero, 1968, 2002). Despite the widespread acknowledgement of temperature’s importance, we are still making strides in understanding the interaction of temperature, physiology and biological communities (Zillig *et al.,* 2021; Brownscombe *et al.,* 2022; McInturf *et al.,* 2022). Rapid environmental change, whether through local anthropogenic forces or global climate change, will undoubtedly alter the temperature dynamics of ecosystems. Such alterations have the potential to affect organisms and their interactions, which can pose difficulties for the management of these changing environments and conservation of resident endangered populations. Triaging the conservation of endangered species becomes more complicated when the management aims of multiple endangered or otherwise protected species conflict (National Research Council, 1995). Thus, reconciling conflicts among endangered species and predicting the impact of management actions upon non-target species are necessary to maximize the utility of limited conservation resources.

Green sturgeon (*Acipenser medirostris*) are a large, anadromous fish species distributed along the west coast of North America. While adult fish may be found across a wide range of latitudes, from the Bearing Sea to All Saints Bay, Mexico (Moyle, 2002), Green sturgeon currently only spawn in the Rogue, Klamath and Sacramento River Watersheds (USA) (Israel *et al.,* 2004; Seesholtz *et al.,* 2015). Juvenile sturgeon then spend the first 6 to 18 months in freshwater before transitioning to estuarine and eventually marine environments (Allen *et al.,* 2009). The three spawning populations are split into northern (Rogue and Klamath River spawners) and southern (Sacramento River spawners) distinct population segments (nDPS and sDPS, respectively), with the latter being listed as threatened under the Endangered Species Act (NMFS, 2006). Hypotheses for poor population performance among the sDPS include environmental drivers such as increasing temperature and salinity intrusion (Rodgers *et al.,* 2019), loss of habitat primarily via water diversion structures (Mora *et al.,* 2009; Steel *et al.,* 2019b) and other anthropogenic changes to the river systems (Poletto *et al.,* 2014, 2015), such as fishing pressure (Moyle, 2002). Resolving these drivers will improve the efficacy of conservation and management actions.

Sympatric with the sDPS of green sturgeon is the critically endangered population of endemic Sacramento River winter-run Chinook salmon (*Oncorhynchus tshawytscha*). These salmon are reliant upon cold-water releases from Shasta Reservoir, which aim to keep river temperatures below 13.3°C to limit mortality among incubating eggs and alevin (USFWS, 1999). Conversely, work by Poletto *et al.* (2018) has demonstrated that green sturgeon growth is strongly temperature dependent, and temperatures below 13°C elicit minimal growth in juveniles (0.02 g day^−1^ for fish acclimated to 11°C). A meta-analysis by Rodgers *et al.* (2019) confirmed that optimal temperatures for all early life stages of green sturgeon are above 14°C (embryos: 14–16°C; yolk-sac larvae: 18–20°C; post-yolk-sac larvae: 18–24°C; juveniles: 15–19°C). Finally, modelling work by Hamda *et al.* (2019) indicated that decreasing the river temperature to benefit winter-run Chinook salmon negatively impacts the growth rate of green sturgeon. This body of work identifies a conservation conflict wherein management actions for one threatened or endangered species may negatively affect the conservation of a second.

Understanding the role temperature plays on the ecophysiology of green sturgeon is important to predicting how this management conflict may impact the recruitment of juvenile green sturgeon. However, the effects of temperature can be mediated or compounded by interacting environmental stressors. Of particular concern for young life stages of green sturgeon is food availability (Lee *et al.,* 2014; Haller *et al.,* 2015; Poletto *et al.,* 2018). The rapid growth of young sturgeon necessitates abundant food resources and Poletto *et al.* (2018) investigated the interaction between rearing temperature and food abundance, finding strong interactive effects between the two stressors. Fish reared at warm temperatures (16 or 19°C) but fed a reduced ration exhibited growth rates similar to fish reared at 11 or 13°C provided a full ration. Furthermore, the growth of fish reared at these colder temperatures appeared unaffected by low food abundance. Given the interacting patterns of temperature and food availability, and ongoing changes in food web dynamics in the San Francisco Bay and Sacramento-San Joaquin Delta (Cloern and Jassby, 2012) paired with the management-induced unseasonably cold temperatures of the northern Sacramento River, we sought to explore the impacts of multiple stressors on aerobic metabolism of green sturgeon.

Aerobic metabolism is a wholistic physiological trait that integrates multiple aspects of thermal physiology (e.g. oxygen uptake, energy catabolism) and is relevant to organism growth, swimming performance (Fry, 1947; Brett, 1971; Pörtner and Farrell, 2008) and potential ecological dynamics such as predation and foraging (Brownscombe *et al.,* 2022; McInturf *et al.,* 2022). A higher aerobic scope (AS) value is theorized to facilitate greater fitness and conditions that reduce an organism’s aerobic capacity are considered detrimental (Farrell, 2016). AS has been widely used to assess the thermal performance curves of fishes (Eliason *et al.,* 2011; Chen *et al.,* 2015; Zillig *et al.,* 2023a), particularly in response to environmental change and conservation challenges (Munday *et al.,* 2009; Raby *et al.,* 2016; Zillig *et al.,* 2023b).

To examine the effects of temperature and feed ration on juvenile green sturgeon physiology, we acclimated fish to two temperature regimes (13 and 19°C) and two feed rations [100% and 40% of optimal feed (Deng *et al.,* 2003; Lee *et al.,* 2014)]. We used aerobic metabolism, sampled across a range of acute test temperatures (11–31°C), to further investigate the dual stressors of rearing temperature and feed restriction on green sturgeon. We hypothesized that feed restriction would reduce metabolic performance, particularly when fish are acclimated to warm temperatures. Furthermore, a decline in aerobic performance associated with feed restriction may be exacerbated by high or low test temperatures based upon whether a fish was acclimated to 13 or 19°C, respectively.

## Materials and Methods

The juvenile green sturgeon used in this experiment were spawned from wild nDPS adults caught on the Klamath River by fishermen of the Yurok Tribe in April 2016. sDPS green sturgeon are not available for research due to their protected status, and so the nDPS was used as a proxy. Adult fish were transported to the Center for Aquatic Biology and Aquaculture at UC Davis where they were spawned in accordance with methodology from Van Eenennaam *et al.* (2001). Eggs were held at 15°C until hatch; afterwards, larvae were reared at 18°C in 815-l circular tanks. Larval fish were provided continuous flow of aerated, non-chlorinated fresh water from a dedicated well. After the development of exogenous feeding [ca. 15 days post-hatch (dph), 0.1 g, 3 cm of total length (*L*_T_); Van Eenennaam *et al.,* 2001], fish were fed continuously ad libitum with semi-moist pellets (Ragen, Inc.; www.rangen.com) until the experiment began.

This experiment utilized at 2 × 2 design with fish experiencing one of two acclimation temperatures (13 or 19°C) and one of two feed rates [100% optimal feed rate (OFR) or 40% of OFR (low feed rate, LFR)]. The LFR ration reflects the observation that wild larval green sturgeon are often found with empty stomachs (Zarri and Palkovacs, 2019) but remain in excess of the maintenance ration (~25%, Haller *et al.,* 2015). Thirty-two-dph fish were randomly placed into one of eight 470-l round tanks initially set to 18°C (*n* = 25 per tank). Two replicate tanks were used for each temperature and feed ration combination. Water temperature was adjusted at a rate of 1°C day^−1^ until acclimation temperatures were reached. Once target temperatures were achieved, ration treatments were initiated. Fish were fed continuously using 24-h feeders, and ration treatments were maintained until the end of the experiment (Table 1, treatment duration).

Feed rates for each treatment group were determined using the OFR model for *Acipenser transmontanus* (Hung *et al.,* 1993, 1995; Deng *et al.,* 2003; Lee *et al.,* 2014) as well as data on growth rates of *A. medirostris* under multiple feed rations at 18°C (Zheng *et al.,* 2015). Feed models were built using data from fish acclimated to 18–23°C (Lee *et al.,* 2014). These rations models reflected both fish mass and temperature and were also used by Poletto *et al.* (2018), and further information regarding their derivation can be found there. To maintain 100% or 40% optimal feed, the feed rates were recalculated every 3 weeks to reflect the increasing size of the fish and updated daily to account for fish mortality or experimental use. Feed was delivered 24 h day^−1^. Feed restriction for both the 13 and 19°C groups began on the same day. Due to the effects of the ration on growth (Poletto *et al.,* 2018), treatment groups entered metabolic experiments at similar sizes but different ages and, therefore, different durations of exposure (Table 1). All groups were held under their treatment conditions for at least 33 days. All protocols and handling procedures were reviewed and approved by the UC Davis Institutional Animal Care and Use Committee (Protocol #18767).

**Table 1:** Fish age, mass, length, condition factor and thermal optima for green sturgeon reared at 13 or 19°C and fed either LFR or OFR

Rearing temperature (°C)	Feed ration	*n*	Fish age (days), *μ* ± SD	Treatment duration (days), *μ* ± SD	Mass (g), *μ* ± SD	Total length (cm), *μ* ± SD	Condition factor			RMR Q10, *μ* ± SD	*T*_OPT_ (°C), *μ* ± SD	AS at *T*_OPT_ (mgO_2_ kg^−1^ min^−1^), *μ* ± SD	*T*_CRIT_ (°C), *μ* ± SD
							Relative *K_n_*, *μ* ± SD	Allometric					
								*K*_A_, *μ* ± SD	*b*, *μ* ± SEM				
13	LFR	33	171 ± 5.6	138 ± 5.62	13.67 ± 2.61^a^	15.1 ± 1.0^a^	0.85 ± 0.07^a^	1.00 ± 0.08^a^	2.66 ± 0.03	2.39 ± 0.16	19.7 ± 0.3^x^	9.86 ± 0.16^y^	36.4 ± 0.8
13	OFR	31	155 ± 6.7	123 ± 6.7	18.03 ± 2.99^b^	16.3 ± 1.0^b^	0.92 ± 0.07^b^	0.85 ± 0.06^b^	2.74 ± 0.03	2.12 ± 0.12	19.7 ± 0.2^x^	9.83 ± 0.16^y^	33.9 ± 0.4
19	LFR	33	104 ± 11.1	72 ± 11.1	13.35 ± 1.55^a^	15.7 ± 0.6^ab^	0.75 ± 0.08^c^	1.32 ± 0.08^c^	2.51 ± 0.03	2.37 ± 0.15	21.7 ± 0.2^y^	8.99 ± 0.13^z^	36.1 ± 0.5
19	OFR	37	78 ± 11.0	46 ± 11.0	12.71 ± 4.64^a^	14.3 ± 1.7^c^	0.85 ± 0.07^a^	1.19± 0.11^d^	2.61 ± 0.02	2.06 ± 0.10	20.3 ± 0.2^z^	9.04 ± 0.13^z^	35.9 ± 0.6

Average fish age and duration of exposure are given at the time of the metabolic trials. Condition factor is reported as both the relative condition factor (*K_n_*) using the length weight relationship in Poletto *et al.* (2018) and as the allometric condition factor (K) with treatment-specific scaling exponent b. Superscript letters (^a, b, c^) indicate significance (*P* < 0.05) between treatment groups determined using an ANOVA. The Q10 of the RMR values was calculated using model results at acute temperatures of 13 and 30°C. The mean *T*_OPT_ of repeated draws from the AS model and the model estimated AS at that temperature. Superscript letters (^x, y, z^) indicate significance between treatment *T*_OPT_ and AS at *T*_OPT_ values. These significance values were assigned via pairwise contrasts of the model estimates of *T*_OPT_ and AS at *T*_OPT_ with significance assigned if >89% of the contrast distribution was above or below 0. Estimated *T*_CRIT_ is given without assignment of significance because the estimated values fall outside the range of temperatures tested during metabolic trails.

### Respirometry

Fish (mass: 13.22 ± 3.26 g, total length: 15.3 ± 1.4 cm; *μ* ± SD, *n* = 166) underwent metabolic trials in one of three 5-l automated swim tunnel respirometers (Loligo, DNK). Two tunnels were controlled using a single computer system, and the third was controlled with a separate system. Water for each swim tunnel system was pumped (1260, Eheim, DEU) from a designated sump into an aerated water bath surrounding each swim tunnel, which overflowed to the sump. Sump water was continually supplied with non-chlorinated fresh water from a designated well and aerated with porous air stones. The temperature of the sump water (and therefore the swim tunnels) was maintained by circulating water through a heat pump (model DSHP-7; Aqua Logic Delta Star, USA) and pumping it back to the sump using a high-volume water pump (PM700, Pondmaster, USA). In addition, each sump contained an 800-W titanium heater (TH-800, Finnex, USA) connected to a thermostatic controller. Water temperature within the swim tunnels was maintained to a precision of ±0.5°C. Swim tunnels and associated sump systems were disinfected with bleach weekly to reduce potential for bacterial growth with the system.

Dissolved oxygen saturation within the swim tunnels was measured using fibre-optic dipping probes (Loligo, DNK), which continuously recorded via AutoResp™ software (version 2.3.0). Oxygen probes communicated to the AutoResp™ via a Witrox-4 oxygen meter (Loligo) for the two-tunnel system and a Witrox-1 oxygen meter for the single-tunnel system. Oxygen probes were calibrated weekly using a two-point, temperature-paired calibration technique. Water velocity of the swim tunnels was quantified and calibrated using a flowmeter (Hontzcsh, DE) and regulated using a variable frequency drive controller (models 4x and 12K; SEW Eurodrive, USA). The velocity (precision <1 cm s^−1^) for each tunnel was controlled remotely using the AutoResp™ programme and a DAQ-M data acquisition device (Loligo). Swim tunnels were surrounded by shade cloth to reduce experimenter disturbance on the fish. Fish were remotely and individually monitored using infrared cameras (QSC1352W; Q-see, CHN) connected to a computer monitor and DVR recorder.

Oxygen consumption rates for both routine and maximum metabolic rates (MMRs) were measured via intermittent respirometry (Brett, 1964). The ratio of fish mass to respirometer volume was 1:376 (±92, *μ* ± SD) A flush pump (DC30A-1230; Shenzhen Zhongke, China) for each tunnel pumped in aerated fresh water through the swim chamber and was automatically controlled via the AutoResp™ software and DAQ-M system. The DAQ-M would seal the tunnel and enable the measurement of oxygen consumption attributable to the fish. Oxygen saturation levels were kept above 80% and restored within 3 min with the influx of fresh, oxygenated water. Due to routine weekly sanitization, controls for background bacterial respiration were not conducted. We compared the routine metabolic rate (RMR) values for fish measured in the first 2 days after sterilization with those measured in the last 2 days and found no significant difference between the two groups (*P* = 0.91).

### Routine metabolic rate

Test fish were fasted for 23.8 ± 2.10 h in 0.5 m × 1.0 m rectangular holding tanks with aerated flow-through water at their acclimation temperature. Faecal matter was never observed within the swimming tunnels supporting that fish were in a post-prandial state during trials. Fish were then transferred into a swim tunnel respirometer between 9:30 am and 4:00 pm to account for differences in time needed to adjust fish to test temperatures. Fish were provided a 30-min period at their acclimation temperature before a brief training swim wherein fish were exposed to a 25 cm s^−1^ current for 30 min followed by 10 min at 45 cm s^−1^. Training swim was done to assess whether green sturgeon would orient and swim against the artificial current. After the training swim, fish were allowed to recover for 1 h before adjusting the temperature of the swim tunnels. The temperature was adjusted at 2°C h^−1^ to one of the swimming test temperatures (seven to nine temperatures ranging from 11 to 31°C, Table 2). Once temperature was achieved, fish were given another 1-h acclimation period, after which RMR data were collected (Verhille *et al.,* 2016; Zillig *et al.,* 2023b). Using intermittent flow respirometry, oxygen data were sampled overnight and into the morning preceding the start of the MMR trial at 08:14 am ± 22 min. Measurement windows ranged in size from 600 to 1200 s to accommodate the influence of temperature on the metabolic rate of the sturgeon. Flush periods were 180 s and the wait period was 120 s. Fish activity was monitored by overhead infrared cameras, and data from fish that were not quiescent were discarded. RMR was calculated by averaging the three lowest RMR values (Poletto *et al.,* 2017).

**Table 2:** Table of fish used in metabolic experiments identified by acclimation temperature, feed ration and metabolic test temperature

Acclimation temperature (°C)	Feed ration	Metabolic test temperature (°C)	Total number of fish used in analysis	Total number of fish trialled
13	LFR	11	4	4
13	LFR	13	6	7
13	LFR	15	4	5
13	LFR	19	4	5
13	LFR	23	5	7
13	LFR	27	6	8
13	LFR	29	4	5
13	OFR	11	4	4
13	OFR	13	4	4
13	OFR	15	5	5
13	OFR	19	4	5
13	OFR	23	4	7
13	OFR	27	5	5
13	OFR	29	5	5
13	OFR	31	0	2
19	LFR	11	4	4
19	LFR	13	5	6
19	LFR	15	3	4
19	LFR	19	5	6
19	LFR	23	5	6
19	LFR	27	5	10
19	LFR	31	6	6
19	OFR	11	4	4
19	OFR	13	4	4
19	OFR	16	4	4
19	OFR	19	4	6
19	OFR	22	4	6
19	OFR	24	4	6
19	OFR	26	4	8
19	OFR	28	4	4
19	OFR	30	4	4

Fish would be discarded for analysis due to non-quiescence during the RMR (15.7%), user error or equipment malfunction (3.0%) or mortality (1.2%).

### Maximum metabolic rate

After completion of the RMR, a fish’s MMR was elicited using a ramped velocity protocol (Jain *et al.,* 1997; Verhille *et al.,* 2016; Poletto *et al.,* 2017; Zillig *et al.,* 2023b). At the start of a swim trial, tunnel speed was increased gradually from 0 to 30 cm s^−1^ over a 2-min period and held there for 20 min. For each subsequent 20-min measurement period, tunnel velocity was increased by 10% up to a maximum of 6 cm s^−1^ per step (~0.5 BL s^−1^). Swimming metabolism was measured by sealing the tunnel for 15 min of each 20-min measurement period followed by 3-min flush period and a 2-min wait period. Impingement on the back screen (>2/3 of body in contact with screen for longer than 3 s) resulted in stopping the tunnel velocity for ~1 min followed by a gradual increase to the original speed over 2 min. A fish was determined to be exhausted if it became impinged twice within the same velocity step. Upon the final impingement, the tunnel impellor was stopped to allow for recovery. The highest metabolic rate measured over a minimum of 5 min at any point during the MMR trial was taken as the MMR and visually inspected for linearity (*R*^2^ ≥ 0.9).

Post-experiment, the tunnel was returned to the acclimation temperature and then fish were transferred to a separate tank and monitored for recovery. Data from trials that suffered mechanical errors (3.0%) or when fish did not recover (1.2%) or were not quiescent during the RMR periods (15.7%) were discarded prior to analysis. After a 24-h recovery period, fish were euthanized via overdose of MS-222 (0.5 g l^−1^) buffered with sodium bicarbonate (0.42 g l^−1^) and salt (6.0 g l^−1^). Measurements for weight (± 0.01 g), fork and total length (± 0.1 cm) were taken. A fish’s AS was calculated as the difference between its RMR and MMR.

### Quantitative analysis

#### Determining metabolic rate

Oxygen concentration was transformed from oxygen saturation data from AutoResp™ using the following equation:

(2) \begin{equation*} \left[{O}_2\right]=\frac{\%{O}_2 Sat}{100}\times \alpha \left({O}_2\right)\times BP \end{equation*}

*%O_2_Sat* is the oxygen saturation percentage reported from AutoResp™; *αO_2_* is the coefficient temperature-corrected oxygen solubility (mgO_2_ l^−1^ mm Hg^−1^); and *BP* is the barometric pressure (mm Hg). Oxygen concentration (milligrammes of oxygen per litre) was measured every second and regressed over time; the coefficient of this relationship (milligrammes of oxygen per litre per second) was then converted to metabolic rate (milligrammes of oxygen per kilogramme per minute, Equation (3)).

(3) \begin{equation*} \mathrm{MR}=R\times V\times{M}^{-1}\times 60 \end{equation*}

*R* is the calculated coefficient of oxygen over time, *V* is the volume of the closed respirometer, *M* is the mass of the fish in kilogrammes and *‘60’* transforms the rate from per second to per minute. An allometric scaling exponent was not incorporated due to similarity in fish sizes (Table 1).

#### Calculating the condition factor

Due to the non-fusiform shape of juvenile sturgeon and the effects of rationing on body morphology, Fulton’s condition factor is not an appropriate measure (Le Cren, 1951; Froese, 2006). We elected to calculate the condition factor using two distinct methods that accommodate either treatment-specific effects on growth (allometric condition factor, *K*_A_) or the non-fusiform body shape of sturgeon (relative condition factor). The first approach calculated *K*_A_ using the equation *K*_A_ = 100*Mass/T_L_*^b^*, where *b* was specific to each treatment group. We used mass data from Poletto *et al.* (2018), which studied the same cohort of juveniles and used the same temperature and ration conditions to calculate *b* for each treatment group. Using this length and mass data, we regressed the natural log of fish mass against the natural log of fish total length. The slope of this length–weight relationship is *b* (Bolger and Connolly, 1989).

We also calculated relative condition *K_n_* = *M*_Ob_/*M*_Pr_ (Neumann *et al.,* 2012), where *M*_Ob_ is the observed mass of a fish and *M*_Pr_ is the predicted mass based upon the fish’s fork length (*L*_F_) and the mass of a reference population using the equation [log_10_(*M*_Pr_) = −1.60117 + 2.4774 log_10_(*L*_F_): Poletto *et al.,* 2018]. The reference population were fed optimal feed and reared at 18°C, although these conditions are arbitrary. Both *K_n_* and *K*_A_ can be interpreted similarly to Fulton’s condition factor with higher values indicating greater body condition.

#### Models

Fish traits (mass, total length and condition factors) were compared using linear models in R (version 4.1.3) with significance assigned if *P* < 0.05. Metabolic traits (RMR, MMR and AS) were also analyzed in R using the Bayesian statistical package *brms* (Bürkner, 2017, 2018), and *ggplot* (Wickham, 2016) was used for visualization. Bayesian metabolic models incorporated uniformed priors and categorical fixed effects for acclimation temperature and feed rate and continuous variables for metabolic trial temperature. Model residuals were visually inspected for normality and homogeneity of variance. The model with lowest Watanabe–Akaike information criterion (WAIC) was selected for investigating treatment differences. Additional fixed effects for fish mass, the condition factor or individual swim tunnel were incorporated as part of model selection. Significance among metabolic traits (MMR, RMR, AS) was evaluated by comparing the overlap of the model-derived posterior distributions among the four treatment groups. Comparing posterior distributions allowed for assessment of significance across the entire temperature gradient. Two treatments were considered significantly different at a given test temperature if the 89% credible interval of the contrast distribution did not overlap zero.

#### ***T***_**OPT**_ **and *T***_**CRIT**_ **calculation**

The thermal optimum (*T*_OPT_) is the temperature at which AS is maximized (Schulte *et al.,* 2011). Values for the *T*_OPT_ and corresponding AS were calculated using 1000 simulated datasets randomly sampled from the posterior distributions of the best-fitting AS model. For each simulated dataset, *T*_OPT_ was calculated by fitting a quadratic equation and calculating the root of the first derivative (Zillig *et al.,* 2023b). The fitted equation was then solved using the *T*_OPT_ value to produce a modelled maximum AS. These two values are reported as means and standard deviations for each treatment group (Table 1, Fig. 2C). We assigned significance of *T*_OPT_ and AS at the *T*_OPT_ by calculating the difference in posterior distribution of each pair of treatments. If 89% of the resulting distribution was above or below 0, then the model estimated values of *T*_OPT_ or AS at *T*_OPT_ were considered significantly different. Using the simulated datasets, we were likewise able to estimate the upper temperature at which AS is zero (*T*_CRIT_). To estimate *T*_CRIT_ from each simulated dataset, we identified the higher of the two temperatures at which the fitted quadratic function equaled zero.

## Results

**Figure 1:**
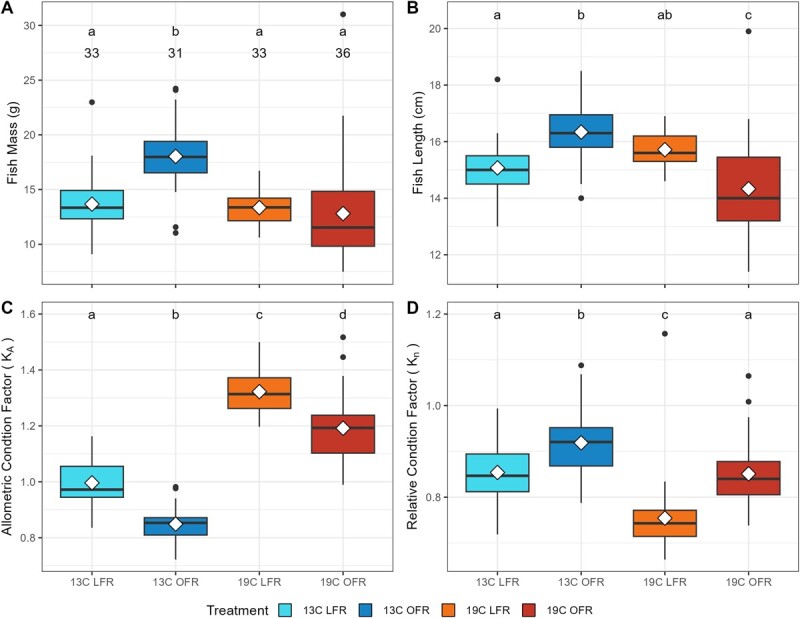
Fish mass (A), total length (B), allometric condition factor (C) and relative condition factor (D) for fish acclimated to 13 or 19°C and fed either low (LFR) or optimal (OFR) rations for >33 days. Boxplots represent median, interquartile range and minimum and maximum values excluding outliers that are represented as points. Means are presented as open diamonds. Letters denote significance (*P* < 0.05) between treatment groups. *N* values for each treatment are above the boxplot in A.

Due to differences in treatment duration (Table 1), the 13°C OFR group exhibited greater mass (18.03 ± 2.99 g) than the remaining treatment groups (collective mean: 13.22 ± 3.26 g). Fish lengths (Fig. 1B) varied among groups from 16.3 ± 0.97 (13°C OFR) to 14.3 ± 1.66 cm (19°C OFR). Temperature and ration also influenced the isometry of the sturgeon, so that treatments varied in their respective proportions; generally, LFR fish exhibited larger heads to smaller bodies yielding a stunted appearance. These variations were not directly measured but can be seen in the incongruence of different condition factor measures. The allometric condition factor (*K*_A_) ranged from 0.85 to 1.32 and was higher among fish reared at 19°C and among LFR fish (*P* < 0.0001; Fig. 1C). The relative condition factor (*K_n_*) ranged from 0.75 ± 0.08 (19°C LFR) to 0.92 ± 0.07 (13°C OFR), with LFR fish having lower *K_n_* than OFR fish (*P* < 0.0001), and with fish reared at 13°C having significantly (*P* < 0.0001) higher *K_n_* than comparably rationed fish reared at 19°C (Fig. 1D).

### Routine metabolic rate

**Figure 2:**
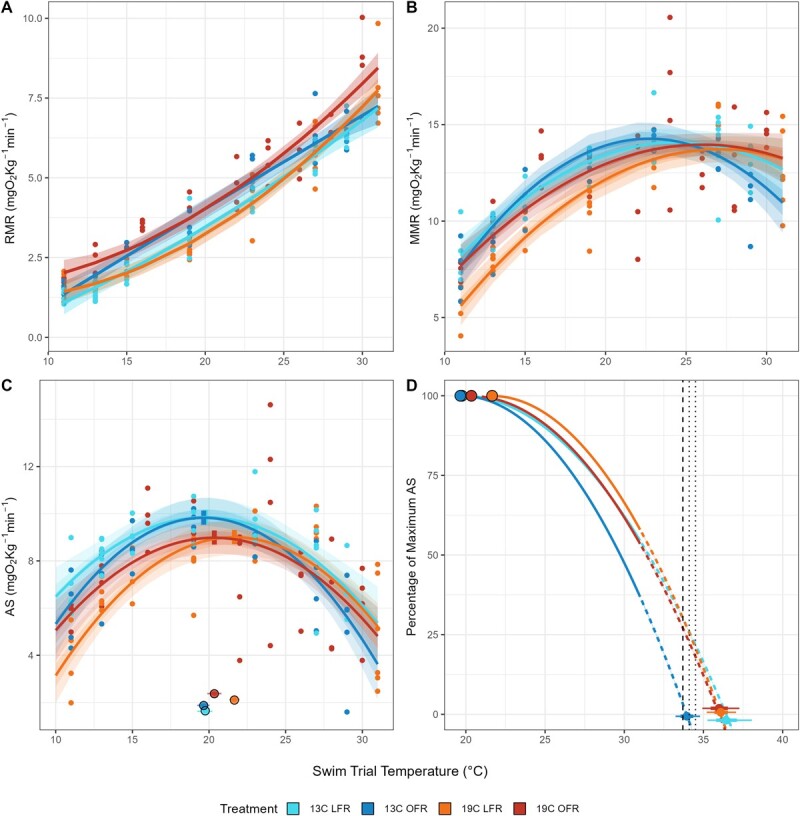
Metabolic performance of green sturgeon acclimated to two temperatures (13 and 19°C) and fed two distinct rations: LFR and OFR. (A) RMR, (B) MMR, (C) AS with ringed points representing the model estimated temperature of *T*_OPT_ and (D) percentage of aerobic capacity available at temperatures above *T*_OPT_. Estimated *T*_CRIT_ for each treatment group is represented as a point (mean) and 95% and 50% credible interval (whiskers) on the *x*-axis. Extrapolated dashed lines indicate data predicted from the model, beyond the upper test temperature of 31°C. Vertical lines indicate measured CT_MAX_ values for green sturgeon reported in Sardella *et al.* (2008), dashed, and Verhille *et al.* (2015), dotted.

Within an acclimation temperature (13 or 19°C), the LFR treatment had significantly lower RMR values across the range of tested temperatures (Fig. 2A). Within a ration group (OFR vs LFR), acclimation temperature did not influence RMR. RMR was modelled as a second-order polynomial of test temperature with interactions of acclimation temperature and feed ration (Supplementary Table S1).

### Maximum metabolic rate

Fish acclimated to 13°C showed no significant effects of rationing on MMR across trial temperatures. Among fish acclimated to 19°C, OFR significantly increased MMR at temperatures below 18°C. MMR was generally lower among fish acclimated to 19°C versus fish acclimated to 13°C, but this effect was only significant among LFR fish and at temperatures <22°C (Fig. 2B). MMR was modelled as a quadratic function of test temperature with an additional interaction between fish mass and feed ration, although neither the interaction nor fish mass alone was a significant predictor (Supplementary Table S2).

### Aerobic scope

Among fish acclimated to 13°C, feed ration did not have a significant effect on AS. Likewise, AS across temperatures was not significantly different among fish in the OFR groups, although acclimation to 19°C generally reduced AS. The AS of the 19°C LFR group when compared to the 13°C LFR group was significantly reduced at temperatures below 20°C. When compared to the 19°C OFR, the 19°C LFR group exhibited reduced AS at temperatures <15°C (Fig. 2C). Variation in AS values was greater among fish acclimated to 19°C. The final model predicted AS as a second-order polynomial function of test temperature with an additional predictor of fish mass. Fish mass was significantly and negatively associated with AS (Supplementary Table S3).

### ***T***_**OPT**_ **and *T***_**CRIT**_

For fish acclimated to 19°C, LFR fish had a higher *T*_OPT_ (21.7 ± 0.2°C) than OFR fish (20.3 ± 0.2°C). Food restriction had no effect on the AS value at *T*_OPT_ among fish acclimated to 19°C (8.99 vs 9.01 mgO_2_ kg^−1^ h^−1^). Fish acclimated to 13°C had a lower *T*_OPT_ and no effect of ration (19.7 ± 0.3°C: 13°C OFR, 19.8 ± 0.2°C: 13°C LFR); however, fish acclimated to 13°C had greater AS at the *T*_OPT_ than fish acclimated to 19°C (Fig. 2C). *T*_CRIT_ values (Fig. 2D) were higher than the highest temperatures at which we tested sturgeon metabolism (31°C) and ranged from 34.0 ± 0.4 (13°C OFR) to 36.5 ± 0.7°C (13°C LFR).

## Discussion

The results of this work highlight the impacts that multiple stressors (temperature and feed restriction) can have on the metabolic capacity of green sturgeon. Feed restriction had little influence on the metabolic capacity of cold-acclimated fish; however, among warm-acclimated sturgeon, feed restriction constrained aerobic capacity at test temperatures below 20°C. As metabolic performance is associated with organismal fitness (Brown *et al.,* 2004; Brownscombe *et al.,* 2022), it is important to understand how environmental factors such as temperature and food availability influence green sturgeon.

### Green sturgeon physiology

Modelled estimate means of RMRs for green sturgeon acclimated to 13°C ranged from 93.9 ± 9.52 mgO_2_ kg^−1^ h^−1^ (LFR, 171 dph) to 117.2 ± 9.83 mgO_2_ kg^−1^ h^−1^ (OFR, 155 dph), which is greater than values from other studies of green sturgeon acclimated to 13°C and of a similar age (72.90 ± 7.33 at 100 dph and 63.36 ± 6.02 mgO_2_ kg^−1^ h^−1^ at 170 dph; Allen and Cech, 2007). Likewise, green sturgeon measured, acclimated and tested at 19°C exhibited lower RMRs (130 ± 7 mgO_2_ kg^−1^ h^−1^) in work by Mayfield and Cech (2004) than RMR values measured in this study for 19°C acclimated fish [176.5 ± 10.2 (LFR) to 223.6 ± 9.83 mgO_2_ kg^−1^ h^−1^ (OFR)]. The higher values reported here may be due to fish being fasted for longer (72 h) in Allen and Cech (2007) as opposed to ~24 h in the present study. Furthermore, the fish used in the present study are smaller (14.23 ± 3.82 g) than the fish used in prior studies (Mayfield and Cech, 2004, 30.3 ± 17.5 g, and (Allen and Cech, 2007, 23.70 ± 1.33 g), and as none of these data were allometrically scaled, we would anticipate higher mass-specific metabolic rates among smaller fish. Our estimates of *T*_OPT_ (19.7–21.7) include the model-derived estimate of the temperature, which bounds maximum feed assimilation (*T*_C_, 20.52°C) estimated by Hamda *et al.* (2019). The congruency between these values supports the assertion that metabolic traits (e.g. AS, *T*_OPT_) can be predictive of overall fish thermal performance.

Poletto *et al.* (2018) found that ration had a smaller influence on the growth of fish acclimated to 13°C. For instance, the average mass of optimally fed fish reared for 63 days at 13°C was only 2.5 g greater than fish on LFR, a 32% increase. When fish were reared at 19°C, optimally fed fish weighed 69.6 g more (a 427.7% increase) than fish fed their reduced ration counterparts, indicating a profound effect of rearing temperature. Similar results were found by Mayfield and Cech (2004) who reared green sturgeon at three temperatures (11, 15 and 19°C) and two rations (50% and 100%) and found that the loss of growth capacity associated with rationing increased with acclimation temperature. In the present study, we repeated the treatment groups of Poletto *et al.* (2018) and found an interaction of temperature and ration among several of the metabolic traits we measured. In both AS and MMR, food availability had little effect upon fish acclimated to 13°C. However, for fish acclimated to 19°C, food restriction reduced MMR and, consequently, AS at test temperature below ca. 22°C. Since fish in this study were fasted prior to metabolic trials and were presumed to be in a post-prandial state, their somatic growth potential may be captured within their AS and MMR. The reduced MMR and AS of the low-feed 19°C fish help explain the pronounced effect observed in Poletto *et al.* (2018).

We elected to test fish of comparable sizes as opposed to similar ages or treatment duration, thereby controlling for pre-treatment rearing condition. All fish started treatment conditions at the same age (32 dph), were acclimated to these conditions for a minimum of 33 days and were maintained under these conditions until they reached testable size. This resulted in some fish (e.g. 13°C OFR fish) being exposed to treatment conditions longer than others. Therefore, differences between groups could reflect differences in the length of treatment exposure. However, the treatment exhibiting the most distinct metabolic physiology is the 19°C LFR group, which experienced conditions longer than the 19°C OFR group but shorter than either the 13°C treatment group. Furthermore, the two 13°C groups exhibited very similar metabolic performance despite an average difference of 14 days in treatment duration. Finally, the effects of these treatments on fish growth, documented in Poletto *et al.* (2018), were observed within 21 days and then consistently remeasured over the following 84 days. This stability in effect supports our assumption that our metabolic results capture the effects of temperature and feed restrictions and are not an artefact of treatment duration.

Responses of metabolism to feed restriction are hypothesized to be advantageous in low-feed conditions (McCue, 2010). In this study, green sturgeon RMR decreased with reduced food availability. This hypometabolic response could be due to energetic tradeoffs forced by feed restriction, such as immune system suppression or reduced energy allocated to somatic growth (Wang *et al.,* 2002; McCue, 2010). Alternatively, different tissues exhibit different metabolic rates (Krebs, 1950; Hulbert and Else, 2000), and so differences in metabolism could be due to a reduction in the percentage of respirating material in fasted fish, as the proportion of the body mass that is made of non-respiring tissues such as cartilage and bone increases relative to well-fed fish, which presumably possess more skeletal muscle. Recent research on congeneric white sturgeon (*A. transmontanus*) found that fish fed a reduced ration (50% of optimal) and acclimated to 15°C exhibited a non-significant reduction in standard metabolic rate (SMR) relative to their well-fed counterparts (V. K. Lo, *unpublished data*). Reduction in RMR for a fish undergoing feed restriction, whether due to physiological trade-offs or simple loss of respiring tissue, allows for greater energy conservation without associated costs in maximum metabolic capacity. Additionally, fish fed reduced rations (LFR groups) maintained their MMR, and therefore, the reduction in RMR associated with reduced rations enabled greater AS. 19°C LFR sturgeon had the highest thermal optimum of any treatment (21.6°C), and while rationing did not influence the thermal optimum of fish acclimated to 13°C, 13°C LFR fish preserved a greater percentage of aerobic capacity at temperatures above their *T*_OPT_ than 13°C OFR fish, a result shared by 19°C LFR fish (Fig. 2D). This increase in aerobic capacity may facilitate fish to ‘catch-up’ should food become available in the environment, as greater aerobic resources can be expended to capture prey and convert it into somatic growth. Supporting this hypothesis is behavioural work on white sturgeon, documenting that starved fish exhibited greater swimming activity than fed fish (Steel *et al.,* 2019a), a possible application of a greater metabolic scope. Furthermore, we found that fish fed a reduced diet have higher *T*_CRIT_ values than their OFR counterparts (Fig. 3B). Although these differences could not be statistically tested, they are consistent with past research on the effect of rationing on green sturgeon thermal maxima (Verhille *et al.,* 2015), whereby green sturgeon on reduced rations elicited higher critical thermal maximum (CT_MAX_) values, although this difference was slight and depended upon statistical approach. Greater thermal physiological performance may facilitate starving fish to pursue energy resources in warmer habitats that conspecifics may typically avoid.

Fish from the 13°C OFR group were of greater mass (18.03 ± 2.99 g, *μ* ± SD) than the other treatment groups (13.22 ± 3.27 g, *μ* ± SD) due to the logistical challenges in conducting metabolic trials. Fish mass was a predictor in the best-fit models for MMR and AS. In the MMR model, it was a non-significant predictor, and in the AS model, mass exhibited a significant and negative relationship with AS, with larger fish having lower AS than smaller fish. Since the 13°C OFR group was larger in size and elicited the highest AS, we do not consider the effect of mass as confounding our results. Instead, differences among size-matched fish may be even more pronounced. Green sturgeon grow rapidly as juveniles (Zheng *et al.,* 2015; Verhille *et al.,* 2015; Poletto *et al.,* 2018), spanning multiple orders of magnitude in mass during their juvenile, freshwater life stage (Allen and Cech, 2007). Furthermore, Allen *et al.* (2006) found that metabolic rate of juvenile green sturgeon was relatively size independent until fish were >400 mm long; the fish used in the present study were all under 200 mm (153 ± 14 mm, *μ* ± SD). We believe any mass-related differences between the 13°C OFR group and others to be marginal.

## Conservation Conflict

The Sacramento River winter-run Chinook salmon are a critically endangered, endemic population of Chinook salmon that elicit a unique migratory phenotype. Adults return to freshwater during the winter months and historically would have migrated to the highest reaches of the Sacramento, Pit and McCloud Rivers. Winter-run adults, unlike other populations of California Chinook salmon, spawn in the early summer and embryos incubate through the summer months, kept cold by high-elevation cold-water springs. Construction of large dams have eliminated 100% of the historical winter-run spawning habitat (Yoshiyama *et al.,* 2001; Quiñones *et al.,* 2015), and adults now spawn in the Sacramento River below Keswick dam, sympatric with the natural spawning and rearing habitat of sDPS green sturgeon. As part of the winter-run management plan, water temperatures are kept unseasonably cold (<13.3°C) throughout the summer months via cold-water releases from the depths of Shasta Reservoir.

As documented by Poletto *et al.* (2018), cold-water temperature greatly limits the growth potential of juvenile green sturgeon. Zarri and Palkovacs (2019) documented that wild caught larval green sturgeon exhibited less-full stomachs when caught in cold waters. While the authors could not attribute this to a change in the food abundance, a change in sturgeon foraging activity or both, it indicates that the performance of wild fish reflects what has been observed in laboratory studies. Modelling work by Hamda *et al.* (2019) assessed the relative performance of green sturgeon versus winter-run Chinook salmon under a variety of different hydrographic conditions (e.g. wet vs dry years). Using a bioenergetic model, they compared the temperature-dependent growth trajectory of green sturgeon in the Sacramento River to the egg-to-fry survival of winter-run Chinook salmon. They concluded that the benefits of slightly warmer water to green sturgeon were outweighed by the larger reduction in survival of salmon embryos. However, their modelling approach did not include aspects of temperature-dependent predation or the influence of feed restriction on predicted green sturgeon growth.

Direct comparison of laboratory-based physiological traits between nDPS green sturgeon and winter-run Chinook salmon clearly indicates sturgeon are more thermally tolerant, consistent with their historical habitat conditions. The CT_MAX_ of green sturgeon acclimated to 18°C and reared in freshwater was 33.7 ± 0.1°C (Sardella *et al.,* 2008), and the CT_MAX_ of green sturgeon exposed to different rations ranged from 34.1 ± 0.1 to 34.5 ± 0.2°C, with fish fed reduced rations eliciting a higher CT_MAX_ (Verhille *et al.,* 2015). Additionally, we were able to conduct metabolic trials up to 31°C, and extrapolation of AS relationships to temperatures where AS equals zero (*T*_CRIT_) is inclusive of reported CT_MAX_ values (Fig. 3B). The CT_MAX_ values as well as maximum metabolic test temperatures for green sturgeon are 4–6°C higher than comparable values for sympatric populations of Chinook salmon (Zillig *et al.,* 2023b). Winter-run Chinook salmon acclimated to 11 and 20°C had CT_MAX_ values of 28.1 and 29.6°C, respectively, and a maximum metabolic test temperature of 24–25°C (Zillig *et al.,* 2023b). Green sturgeon also elicited greater *T*_OPT_ of 19.7–21.7°C than winter-run salmon (19.2°C when acclimated to 11°C and 18.0°C when acclimated to 20°C). As a final indication of differing thermal physiology, green sturgeon are documented to grow rapidly at temperatures ranging from 19 to 24°C (Allen *et al.,* 2006), whereas winter-run Chinook salmon exhibited minimal growth capacity when acclimated at 20°C (Zillig *et al.,* 2023b).

While the fundamental thermal physiology of green sturgeon may be considerably more eurythermal than sympatric Chinook salmon, when evaluating conditions for conservation, the ecological implications of thermal regimes must be considered (Zillig *et al.,* 2021). Climatic and anthropogenic changes have resulted in increased water temperatures on the west coast of North America (Cloern *et al.,* 2011; Diffenbaugh *et al.,* 2015). The reduced aerobic capacity of warm-acclimated green sturgeon (relative to cold-acclimated sturgeon) may pose ecological challenges for rearing juveniles. Under warm-water conditions, juvenile green sturgeon grow rapidly but are very sensitive to feed limitations (Poletto *et al.,* 2018). While a warmer habitat (with abundant food) may facilitate rapid growth, a cold habitat may mitigate the risks of reduced food availability.

Environmental temperature also impacts trophic dynamics. Warmer environments may increase exposure of juvenile sturgeon to non-native, warm-water predators such as largemouth bass (*Micropterus salmoides*) and striped bass (*Morone saxatilis*). Baird *et al.* (2020) found that predation upon green sturgeon by largemouth bass and striped bass was strongly dependent upon prey size, and a similar result was documented among white sturgeon (A. E. Steel, *unpublished data*). Therefore, if food is abundant, fast growth at warm temperatures may offer juvenile sturgeon a refuge from predation. However, in the instance of limited food, warm temperatures may expose juveniles to increased predation pressure but with an impaired capacity to grow. In contrast, cooler water may reduce the potential for sturgeon growth but also suppress predator abundance and proclivity to predate. Additionally, temperature has an influence on the swimming capacity of juvenile sturgeon (Allen *et al.,* 2006; Deslauriers and Kieffer, 2012). Changes in the relative performance in swimming capacity have been linked to changes in trophic interactions between largemouth bass and juvenile Chinook salmon (McInturf *et al.,* 2022), and should be expected that artificially cold temperatures may influence the biotic interactions among green sturgeon and their environment. Therefore, reconciling the conservation conflict between green sturgeon and Sacramento River winter-run Chinook salmon will require understanding the effect of temperature on both organism physiology and its ecology.

Understanding the potential for conservation conflict between green sturgeon and sympatric salmonids will require an ability to deduce fish condition among wild rearing juveniles. Unfortunately, common approaches to quantifying condition factors are challenged by sturgeon morphology. Body shape in sturgeon has been shown to be influenced by both rearing temperature and food availability, with fish exposed to reduced rations becoming stunted in their length (Poletto *et al.,* 2018). In fisheries literature, fish body condition is often used to assess the general health of a fish or population (Froese, 2006). Body condition can be calculated using several different approaches, typically transformations of length and weight data, with the most common being Fulton’s condition factor (*K*_f_ = *W*/*L*^3^). However, this approach may be a poor fit for fish that do not grow isometrically in regard to the cube law (Le Cren, 1951; Froese, 2006). Therefore, we calculated the condition factor in two different ways hypothesized to be more tolerant of differences in body morphology among treatments: using treatment-specific estimates of allometry (*K*_A_) and the relative condition factor (*K_n_*). Our measurements of *K*_A_ did capture the morphometric differences among treatments. Fish on reduced rations develop more stunted body morphology, and therefore, calculated *K*_A_ is higher (Fig. 1C, Table 1). This raises a concern for management, which commonly uses condition factors to judge fish fitness under the assumption that a higher condition factor is better. If fish under poor conditions (LFR) can elicit higher measured condition factors, then it requires a revaluation of how sturgeon fitness can be assessed from body measurements. Poletto *et al.* (2018) used *K_n_* to describe intuitive differences in sturgeon condition (e.g. fish fed optimal rations had higher condition than fish on reduced ration), and our calculation of *K_n_* in the present study returned intuitive patterns of well-fed fish having higher condition factors but exposed a counter-intuitive effect of acclimation temperature. Use of *K_n_* indicates that the 13°C LFR and the 19°C OFR group exhibit similar body condition, a confusing result considering that 19°C OFR would be considered a ‘better’ rearing environment (Mayfield and Cech, 2004; Rodgers *et al.,* 2019). Furthermore, *K_n_* has several drawbacks. The first is the requirement of a carefully measured reference population, which often precludes the application of *K_n_* across studies or among wild-rearing fish. The second is the influence of time on *K_n_*; Poletto *et al.* (2018) found that the relative condition factor was strongly influenced by the age of juvenile sturgeon, alongside rearing temperature and food availability. Given the mixed effects of age (V. K. Lo, *unpublished data*), ration and temperature (Poletto *et al.,* 2018) on sturgeon morphology and the challenges of identifying and applying an appropriate reference set, calculating meaningful *K*_A_ or *K_n_* values for green sturgeon captured from the field is limited. To better assess sturgeon condition, a novel transformation of sturgeon mass and body morphology to determine fish condition is needed.

A final concern for management of green sturgeon could be differences between the nDPS and the threatened sDPS. Fish used in this study are from the nDPS of green sturgeon. It is possible that metabolic relationships between acclimation temperature and metabolism are different for the sDPS. Variation in metabolic capacity among populations has been previously observed in salmonids (Eliason *et al.,* 2011; Zillig *et al.,* 2023b) but is unstudied in sturgeon. Comparative studies between the nDPS and sDPS should be conducted to establish whether the two populations exhibit different thermal physiologies that may reflect juvenile rearing habitat. It should be noted that both DPSs of green sturgeon are sympatric with populations of Chinook salmon. In the Klamath Basin (USA) in particular, several dams are slated for removal in 2023–2024, re-opening large portions of the watershed to anadromous fishes. Studying how these two sympatric species respond to the opening of new habitat will be important for discerning what the shared habitat usage of the sDPS green sturgeon and winter-run Chinook salmon was historically.

## Conclusion

Our results indicate that the effects of feed restriction on metabolic capacity are primarily observed among fish acclimated to warmer temperatures (19°C) and acutely tested at cold temperatures (<16°C), indicating that the thermal threat to this species may be primarily from cold-water temperatures, although the risk of environmental warming to detrimentally impact the ecology of green sturgeon (i.e. increase predation pressure) must be robustly understood. Reduced metabolic performance and growth may make green sturgeon more prone to predation and perhaps less capable of traversing hydraulic challenges. The sympatry of threatened green sturgeon with endangered salmonids that need cold water produces a conservation conflict. Further work studying the impacts of environmental conditions (e.g. temperature, dissolved oxygen, salinity) on ecological interactions of green sturgeon will be necessary to determine how salmonid-focused management actions will influence this species.

## Supplementary Material

Web_Material_coae021
