# Supplementary material for: Effects of acclimation temperature and feed restriction on the metabolic performance of green sturgeon
Source: Conserv Physiol. 2024 May 6;12(1):coae021. doi: 10.1093/conphys/coae021 (PMC11113080; doi:10.1093/conphys/coae021)
Supplement: Web_Material_coae021 [file web_material_coae021.docx]

Supplemental Material

Table S1: Model output estimates for Routine Metabolic Rate (RMR) of Green Sturgeon reared at two acclimation temperatures (ACC_TEMP_FAC: 13 or 19°C) and two feed rates (FEED_RATE: LFR or OFR). Additional predictors included the test temperature (TEST_TEMP) and if a model used a quadratic fit then an additional test temperature parameter (ITEST_TEMP2). Other fixed effects were mass (MASS), Fulton’s condition factor (COND_FAC) and an allometrically scaled condition factor (STURG_COND_FAC), as well as the specific swim tunnel (Fixed effects, TUNNEL_IDB, TUNNEL_IDC). The model with the lowest WAIC (Model #1) was selected for further analysis. Observations are the number of participant fish.


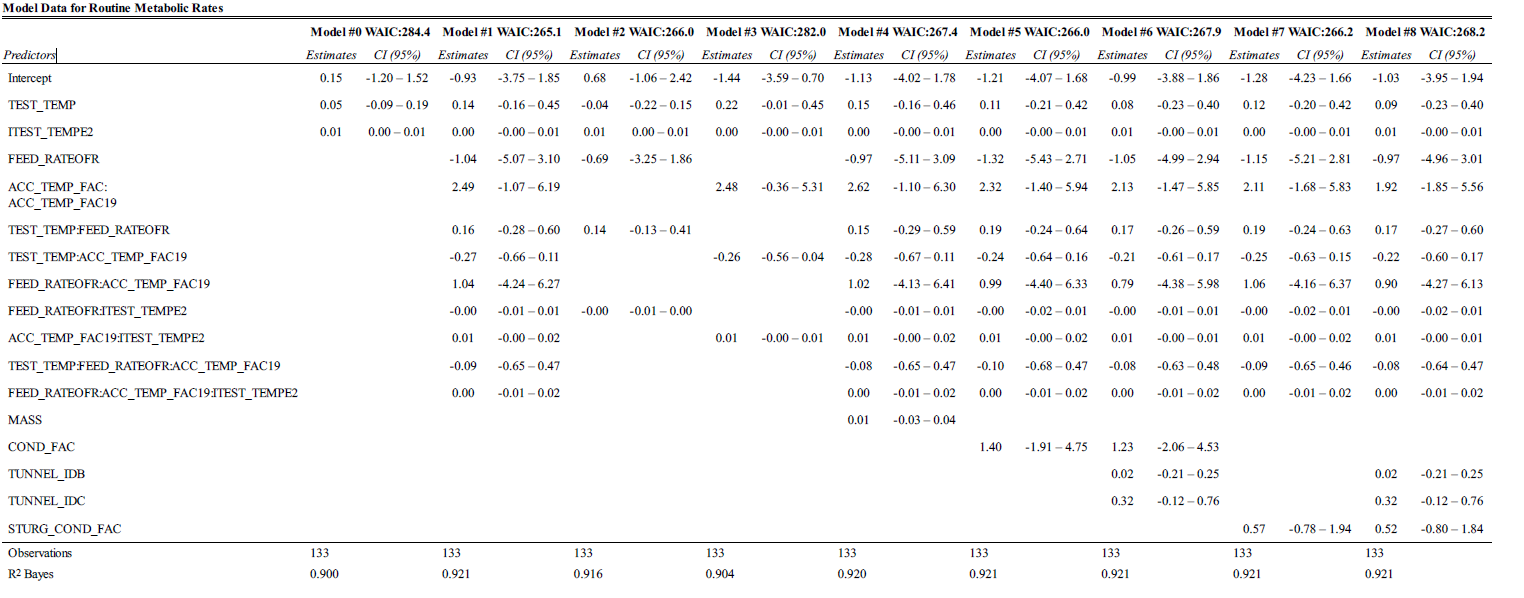


Table S2: Model output estimates for Maximum Metabolic Rate (MMR) of Green Sturgeon reared at two acclimation temperatures (ACC_TEMP_FAC: 13 or 19°C) and two feed rates (FEED_RATE: LFR or OFR). Additional predictors included the test temperature (TEST_TEMP) and if a model used a quadratic fit then an additional test temperature parameter (ITEST_TEMP2). Other fixed effects were mass (MASS), Fulton’s condition factor (COND_FAC) and an allometrically scaled condition factor (STURG_COND_FAC), as well as the specific swim tunnel (Fixed effects, TUNNEL_IDB, TUNNEL_IDC). The model with the lowest WAIC (Model #9) was selected for further analysis. Observations are the number of participant fish.


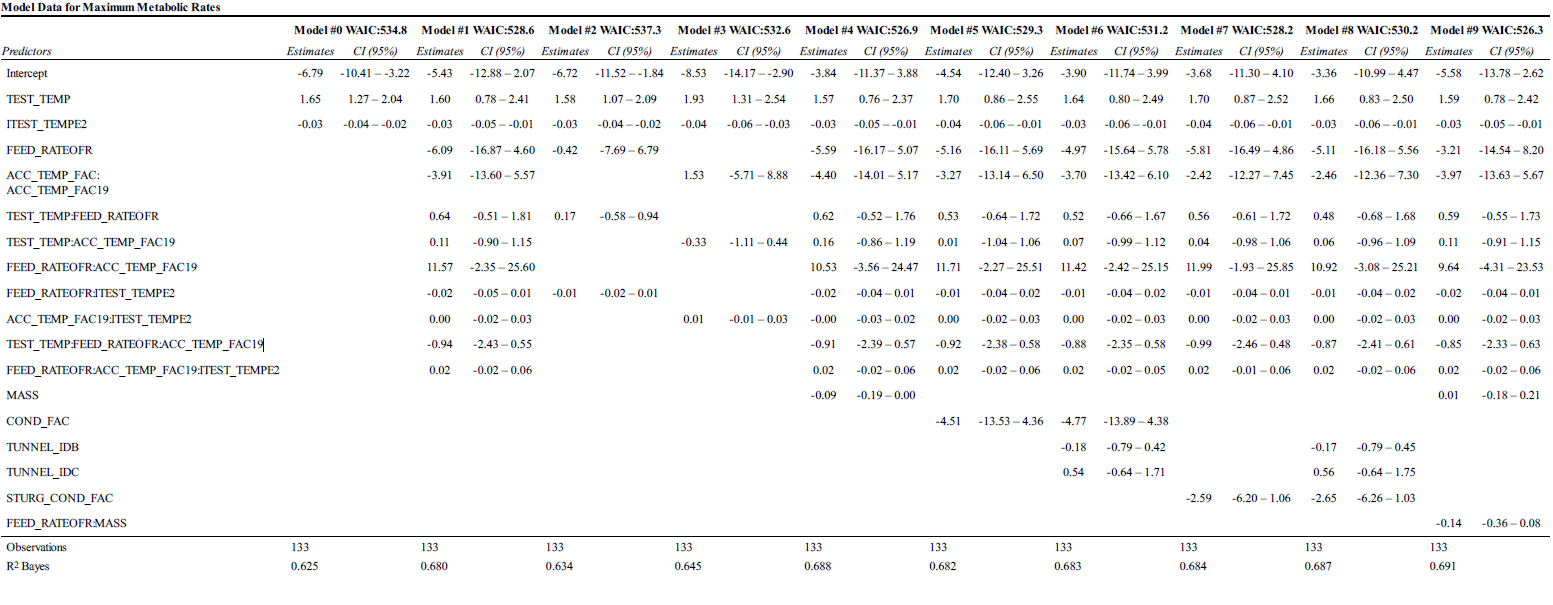


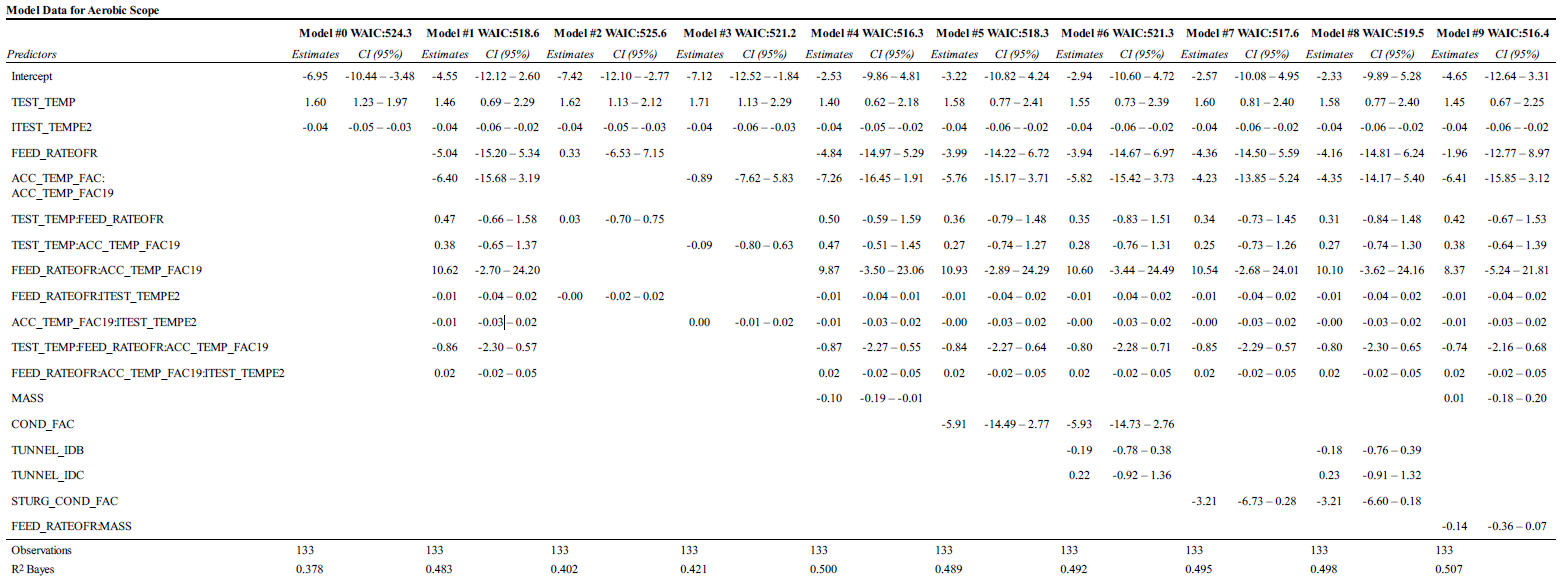


Table S3: Model output estimates for Aerobic Scope (AS) of Green Sturgeon reared at two acclimation temperatures (ACC_TEMP_FAC: 13 or 19°C) and two feed rates (FEED_RATE: LFR or OFR). Additional predictors included the test temperature (TEST_TEMP) and if a model used a quadratic fit then an additional test temperature parameter (ITEST_TEMP2). Other fixed effects were mass (MASS), Fulton’s condition factor (COND_FAC) and an allometrically scaled condition factor (STURG_COND_FAC), as well as the specific swim tunnel (Fixed effects, TUNNEL_IDB, TUNNEL_IDC). The model with the lowest WAIC (Model #4) was selected for further analysis. Observations are the number of participant fish.
